# Variability in Clinical Response to Steroids in Granulomatous Mastitis: A Report of Two Cases

**DOI:** 10.7759/cureus.93940

**Published:** 2025-10-06

**Authors:** Karla V Corona-López, Monserrat Sánchez-Romero, Valeria B Fernández-Rodríguez, Ana K Barba-Rojas, Alejandro Rendón-Molina

**Affiliations:** 1 Medicine, Universidad Nacional Autónoma de México, Mexico City, MEX; 2 Research, Instituto Nanional de Perinatología, Mexico City, MEX; 3 Gynecology, Instituto Nacional de Perinatología, Mexico City, MEX

**Keywords:** anti-inflammatory agents, glucocorticoids, granulomatous mastitis, prednisone, treatment outcome

## Abstract

Idiopathic granulomatous mastitis (IGM) is a rare, benign, chronic inflammatory breast disease that primarily affects premenopausal women, often in the postpartum period or after breastfeeding. Its nonspecific clinical and imaging presentation, frequent overlap with infectious and neoplastic conditions, and variable therapeutic response make both diagnosis and management challenging.

This report aims to describe the clinical characteristics, diagnostic approach, and corticosteroid response in two histologically confirmed cases, highlighting differences in therapeutic outcomes and the relevance of individualized regimens.

Both patients presented with chronic inflammatory breast lesions unresponsive to antibiotics and nonsteroidal anti-inflammatory drugs (NSAIDs), requiring corticosteroid therapy for disease control. One case showed partial response with frequent relapses despite prolonged treatment, whereas the other achieved complete remission with a low-dose, tapered regimen and no recurrences.

Comparative analysis and literature review suggest that disease extent and inflammatory phenotype significantly influence responsiveness, supporting early initiation of prednisone at 0.5-1 mg/kg/day with individualized tapering, and consideration of immunosuppressants in recurrent or steroid-dependent cases. Intralesional corticosteroids may be effective in localized disease, while surgery should be reserved for refractory presentations due to higher recurrence rates. These findings reinforce the role of systemic corticosteroids as first-line therapy for IGM while emphasizing the need for tailored treatment strategies and the generation of local evidence to optimize outcomes, particularly in resource-limited settings.

## Introduction

Idiopathic granulomatous mastitis (IGM) is a rare, benign, chronic inflammatory disease of the mammary gland. It primarily affects premenopausal women, often in the context of the puerperium or following breastfeeding. Clinically, it may mimic common breast infections or even inflammatory breast cancer, making initial diagnosis particularly challenging [1]. Given that idiopathic granulomatous mastitis frequently affects women in the postpartum period, therapeutic strategies must also consider breastfeeding safety. Corticosteroids such as prednisone are generally considered compatible with breastfeeding in low-to-moderate doses; however, prolonged or high-dose regimens may still represent a challenge, requiring individualized planning [2].

The clinical importance of reporting IGM cases lies in their low prevalence, non-specific presentation, and often relapsing course, which together complicate their management. Although no standardized treatment protocol exists, available therapeutic strategies include nonsteroidal anti-inflammatory drugs (NSAIDs), antibiotics, systemic corticosteroids, immunosuppressants, and surgery. Emerging therapies such as intralesional steroid administration are still under investigation regarding their efficacy [3].

In the two cases presented here, diagnosis was hindered by atypical clinical progression, poor response to conventional antibiotic and anti-inflammatory therapy, and inconclusive imaging findings. In one case, the disease manifested bilaterally and relapsed repeatedly, with multiple abscesses and drainage, and no improvement until corticosteroid therapy was initiated. In the other, a suspected but later ruled out association with tuberculous mastitis added complexity to the diagnostic process and delayed definitive treatment. Both cases demonstrate that without prompt and sustained steroid use, clinical improvement would not have been achieved [2].

The aim of this report is to describe the clinical characteristics, diagnostic challenges, and therapeutic response in two cases of idiopathic granulomatous mastitis managed at the Instituto Nacional de Perinatología between March 2023 and April 2025, highlighting the crucial role of systemic corticosteroids in disease resolution and thereby strengthening clinical understanding, particularly in settings with suspected infectious or atypical etiology.

## Case presentation

Case one: torpid evolution and partial response to steroids

A 40-year-old female, G4 C2 P2, with a history of grade II obesity and uterine fibroids, and no autoimmune background, presented in October 2023 with severe right breast pain (visual analogue scale (VAS) 8/10) and progressive inflammation. Physical examination revealed a non-adherent mass and a mobile axillary lymph node. Initial anti-inflammatory management showed no response; antibiotics were escalated, and a spontaneously draining abscess was later documented.

By January 2024, the clinical picture extended to the left breast, with pain, pruritus, and induration. A 62 × 40 mm mass was documented. Biopsy revealed a xanthogranulomatous inflammatory process with reactive epithelial atypia. Oral prednisone at 5 mg/day was initiated, increasing to 20 mg/day in January 2025 due to persistent inflammatory lesions and symptoms. Since April, she has been maintained on 10 mg/day.

Throughout this period, the patient required multiple antibiotic regimens (dicloxacillin, trimethoprim/sulfamethoxazole (TMP/SMX)) and ambulatory drainages in both breasts. Clinical response has been partial, with intermittent symptomatic improvement but frequent recurrences, particularly in the left breast. The most recent episode occurred in April 2025, presenting as spontaneous drainage from an indurated area. This clinical course suggests possible corticosteroid resistance. At the time of the last follow-up, the patient was asymptomatic, under treatment with prednisone 10 mg/day and close surveillance.

Case two: clinical control with low-dose steroids and no relapses

A 47-year-old female, G3 C3, with grade III obesity, uterine fibroids, and chronic venous insufficiency, presented in March 2023 with a mass and pain in the left breast. She was initially treated as a breast abscess with antibiotics, and a core needle biopsy reported fibrocystic mastopathy. Due to persistent symptoms, a second biopsy was performed, confirming chronic granulomatous mastitis with negative special stains (Ziehl-Neelsen, Grocott, PAS staining).

In October 2023, treatment with oral prednisone 5 mg once daily was initiated, accompanied by dicloxacillin for four weeks. The patient showed progressive improvement, documented by imaging studies reporting a reduction in fluid collections and resolution of the inflammatory process (Breast Imaging Reporting and Data System (BI-RADS) 2-3). By January 2024, she was clinically asymptomatic.

Over the following months, the steroid dose was gradually tapered to 2.5 mg orally every 48 hours, achieving complete withdrawal in June 2024. No relapses or need for additional antibiotics were documented. In January 2025, the patient was discharged due to sustained clinical remission. This case stands out for its excellent response to low-dose glucocorticoid therapy, without requiring escalation to immunosuppressive agents.

**Figure 1:**
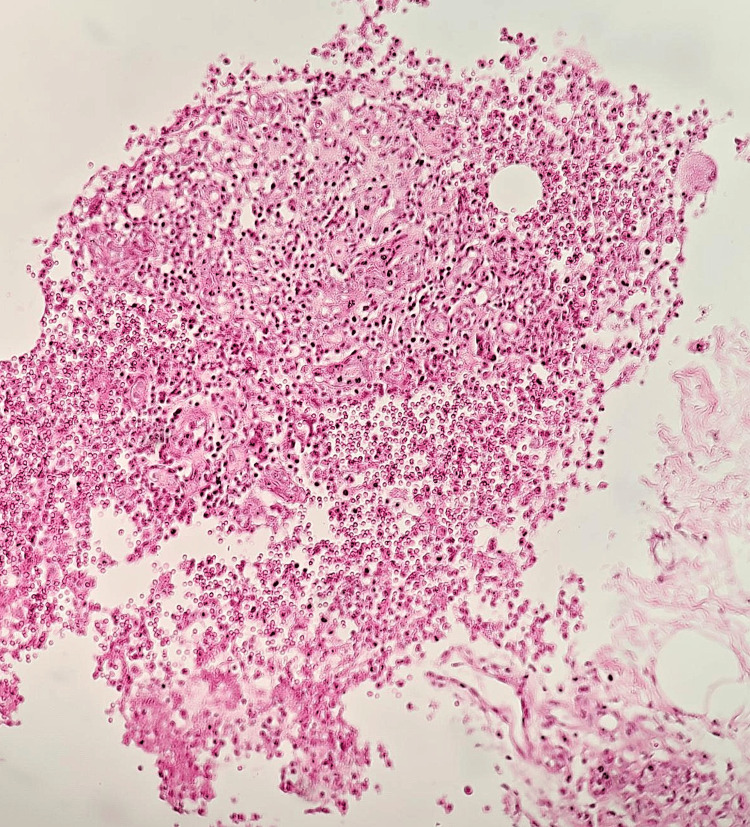
Case one histological section showing mixed inflammatory infiltrate with neutrophil-rich microabscesses and evolving granulomas, consistent with suppurative granulomatous mastitis

**Figure 2:**
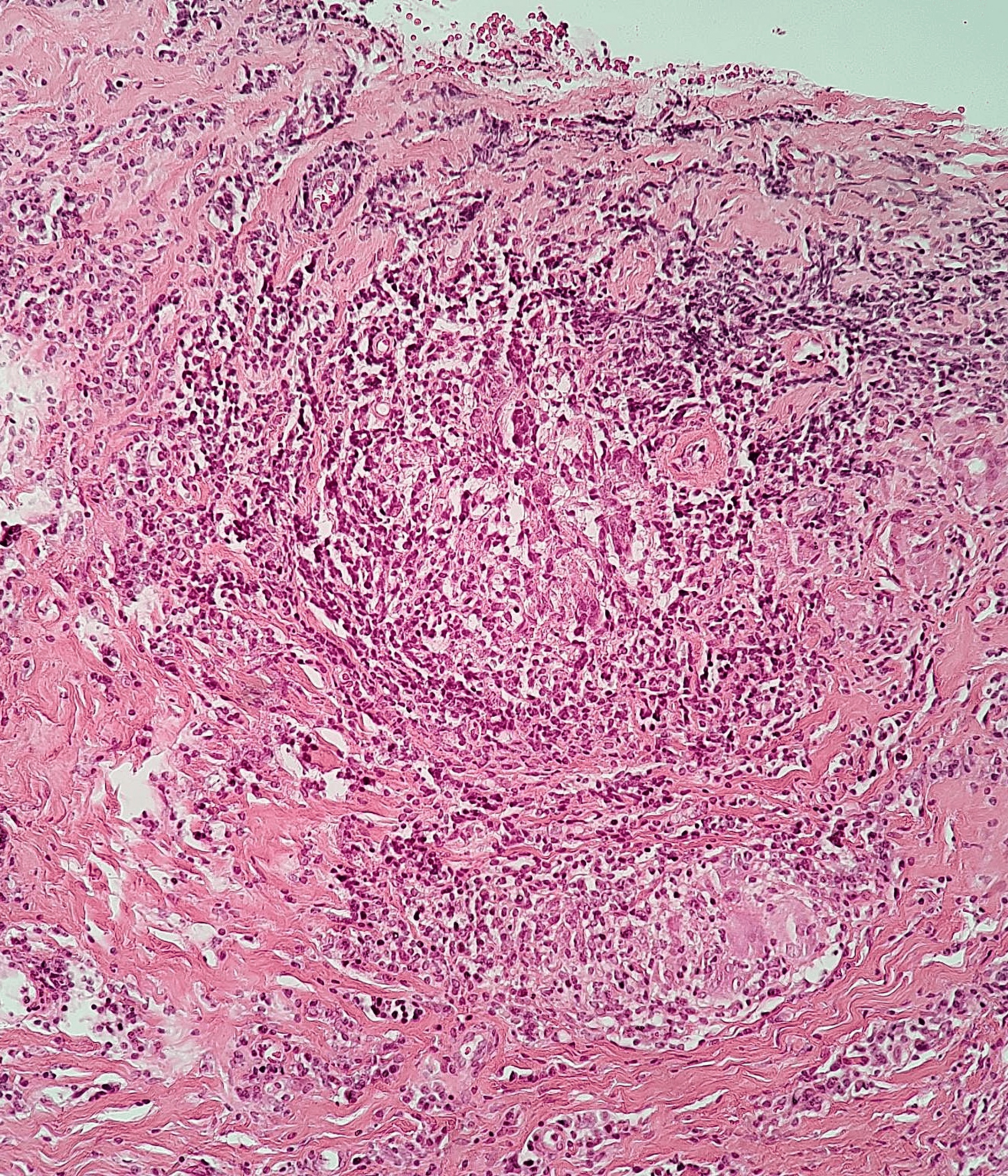
Case two representative histological section showing a well-formed non-caseating granuloma with epithelioid cells, multinucleated giant cells, and surrounding lymphocytic inflammation

Clinical and histological findings of both cases are summarized in Table 1, accompanied by representative histological images illustrating granulomatous inflammation and cellular features from each patient (Figures 1, 2).

**Table 1:** Comparative evolution and steroid response in two patients with granulomatous mastitis

Variable	Patient one	Patient two
Age at diagnosis	40 years	47 years
Comorbidities	Obesity grade II, uterine fibroids	Obesity grade III, uterine fibroids, chronic venous insufficiency
Affected side	Initially right breast, later bilateral	Left breast only
Diagnosis by biopsy	Xanthogranulomatous mastitis with reactive atypia	Chronic granulomatous mastitis, negative stains
Initial dose	Prednisone 5 mg PO every 24 hours	Prednisone 5 mg PO every 24 hours
Treatment course	Increased to 20 mg in January 2025 due to recurrences; tapered to 10 mg	Gradual taper: 5 mg → 2.5 mg every 48 hours; discontinued in June 2024
Total duration of steroid therapy	>1 year (ongoing)	<8 months
Clinical response	Partial, with frequent relapses and need for drainage	Sustained complete response
Need for dose adjustment due to relapse	Yes – increased to 20 mg, then adjusted to 10 mg	No – progressive tapering without flares
Suggested escalation	Yes – candidate for immunosuppressants (e.g., methotrexate)	Not required
Current status	Active disease, on 10 mg PO	Discharged from follow-up since January 2025

## Discussion

Idiopathic granulomatous mastitis (IGM) represents a clinical challenge due to its chronic course, non-specific presentation, and variable therapeutic response. In the absence of standardized guidelines, its approach and management have been based on observational studies and individualized decisions, as demonstrated in the two cases presented [1-3].

From a pathophysiological standpoint, several hypotheses have been proposed to explain its origin, including autoimmune, infectious, hormonal mechanisms, and alterations in the breast microbiome [4]. The autoimmune hypothesis is the most widely accepted, supported by similarities with other granulomatous diseases such as thyroiditis or prostatitis, and by the favorable response to corticosteroids [4]. Associations with breastfeeding have also been described, attributed to increased permeability of the lactiferous ducts due to chemical or physical stimuli, although these mechanisms are still under investigation. Hyperprolactinemia is another proposed etiological factor and has justified the use of bromocriptine in selected cases [1].

Clinically, IGM presents with pain, inflammatory nodules, erythema, and occasionally abscesses or fistulas. Although typically unilateral, bilateral forms have been reported in 1-8% of cases [1]. Its course is chronic, with frequent recurrences, and it often requires surgical incision or drainage due to poor response to conventional antibiotic therapy [1,4].

Differential diagnosis

IGM is a benign inflammatory breast disease that poses a considerable diagnostic challenge due to its non-specific clinical and imaging features. Given its low prevalence and the absence of universal diagnostic criteria, IGM is largely a diagnosis of exclusion, based on the integration of clinical findings, imaging studies, and especially histopathological analysis [5]. Below are the main conditions to consider in the differential diagnosis of IGM.

Breast Abscess

Breast abscess, of infectious etiology, is a common cause of acute breast inflammation. Clinically, it can mimic IGM, presenting with pain, erythema, a palpable mass, and fever [3]. However, it typically responds well to antibiotics and drainage. On imaging, it appears as a well-defined anechoic collection, in contrast to the irregular hypoechoic lesions seen in IGM [3]. A positive culture and the presence of purulent necrosis are distinguishing features of abscesses.

Tuberculous Mastitis

Tuberculous mastitis (TM) should be considered particularly in endemic regions or immunocompromised patients. TM has a specific infectious etiology, with distinctive histological findings such as caseating necrosis and acid-fast bacilli on Ziehl-Neelsen staining [6]. In contrast, IGM presents with non-caseating granulomas and no evidence of mycobacteria. Since the treatment of TM requires antituberculous therapy, accurate differentiation is essential [3].

Inflammatory Breast Carcinoma

Inflammatory breast carcinoma represents one of the most relevant and complex differential diagnoses for IGM, due to the significant overlap in clinical and radiologic features. Studies indicate that in up to 50% of IGM cases, the initial suspected diagnosis is breast cancer, given the severity of clinical presentation and imaging findings [7,8]. On mammography or ultrasound, IGM may present as a spiculated, hypoechoic lesion with irregular margins or architectural distortion-features corresponding to BI-RADS categories 4 or 5. The literature emphasizes the need for histopathological confirmation [3].

Benign Breast Tumors

Benign tumors, such as fibroadenomas or adenomas, may be mistaken for IGM when presenting as palpable masses. Clinically, benign tumors are typically painless, mobile, well-circumscribed, and without inflammatory signs. Biopsy is essential, as cytology has low sensitivity for IGM (<20%) and may be confused with reactive processes in complex benign tumors [8].

Other Entities

Less common conditions to consider include mammary sarcoidosis, foreign body reactions (from implants or trauma), fungal infections (e.g., histoplasmosis), duct ectasia, and rheumatologic diseases such as granulomatosis with polyangiitis, giant cell arteritis, or polyarteritis nodosa.

It is important to note that various studies have reported a higher incidence of IGM in Hispanic and Latin American women compared to Caucasian or African American women [5,9].

Evidence of steroid management in granulomatous mastitis 

The main objectives of steroid treatment are to control active inflammation, prevent recurrences, and reduce sequelae such as persistent pain and breast fibrosis, which negatively impact patients' quality of life [10]. Although spontaneous resolution has been documented in up to 50% of mild cases within a period of 6 to 24 months, expectant management is rarely adequate for moderate or severe symptoms. In these cases, the rational use of NSAIDs and antibiotics may offer temporary relief in the presence of superinfection, but they do not replace immunosuppression with steroids [1].

In the clinical cases analyzed, the use of prednisone was crucial for achieving symptom control. Patient one started with 5 mg daily, with progressive escalation to 20 mg/day due to frequent relapses. She was subsequently titrated to 10 mg daily, without achieving complete remission. In contrast, patient two followed a more conservative regimen with progressive adjustments and complete discontinuation at month nine, achieving sustained remission. In both patients, response was assessed by clinical improvement (pain, suppuration, resolution of abscesses) and imaging findings (reduction of collections and residual fibrosis).

Clinical experience and the literature agree that systemic steroids constitute the first-line therapy for moderate to severe IGM, with response rates of 70-80%, although recurrences are reported in 30-40% [1,11]. The recommended starting dose of prednisolone ranges from 0.5 to 1 mg/kg/day, with progressive reduction over at least 6 to 12 weeks [5]. This raises the question of whether the regimen used in patient one was insufficient. The trial by Montazer et al. demonstrated that a stepped regimen (50-25-12.5-5 mg for two months) achieved a higher remission rate (93% vs. 53%) and lower recurrence (0% vs. 37.5%) than a sustained low-dose regimen, suggesting that an aggressive initial approach could prevent early recurrences in more severe cases [12].

This finding is even more relevant considering that patient one presented with a bilateral form, a rare clinical manifestation documented in only two of 41 patients in a 10-year retrospective study [13]. This more extensive and symmetrical presentation could represent a more inflammatory phenotype or even a more resistant variant of the disease's clinical spectrum, which would explain its poor response to conventional regimens. The literature has suggested that bilateral forms or those with multiple affected areas often require higher doses and prolonged immunosuppression regimens to prevent relapse [1]. Patient two, with a unifocal presentation and less intense symptoms, achieved remission with a less aggressive regimen. This contrast suggests that the inflammatory phenotype and extent of breast involvement significantly modulate the response to immunosuppressive treatment.

This discrepancy also highlights that demographic factors such as age or obesity grade are not necessarily decisive predictors of therapeutic outcome in idiopathic granulomatous mastitis. Instead, recent studies indicate that the extent of breast involvement and the presence of bilateral or recurrent lesions are more closely associated with poor steroid responsiveness [13]. In our series, case one showed a bilateral and relapsing course with abscess formation, features commonly linked to resistance and frequent relapses, whereas case two exhibited a localized, unifocal presentation that allowed complete control with a low-dose tapered regimen. Furthermore, although obesity represents a relevant comorbidity for long-term metabolic risks, its impact on steroid response in IGM has not been consistently demonstrated [7]. These observations support the notion that the initial inflammatory burden and disease extent are more decisive than demographic characteristics in determining therapeutic success. Therefore, personalizing the therapeutic regimen is vitally important, avoiding the indiscriminate application of standard regimens that may be insufficient in some cases or excessive in others. The initial inflammatory burden, lesion size, and the presence of collections should guide both the initial dose and the rate of steroid tapering.

A central aspect in the management of IGM is the recurrence rate after steroid discontinuation or reduction. In a cohort study of 505 patients, it was found that those treated with corticosteroids alone had a 28.8% recurrence rate, significantly higher compared to those receiving immunosuppressants such as azathioprine, either as monotherapy (4.7%) or in combination with steroids (9.1%) [14]. These data reinforce the notion that combined immunosuppression not only enhances remission but also prolongs its duration.

Methotrexate has also demonstrated efficacy as a steroid-sparing therapy. In a Latin American series, three patients achieved complete remission with a regimen of prednisone induction and MTX maintenance, without serious adverse events [15]. Furthermore, other studies have indicated that MTX allows for more rapid reduction of steroid doses, with a lower risk of side effects such as hyperglycemia, weight gain, or insomnia, which significantly improves therapeutic adherence.

On the other hand, early surgical treatment, although tempting for persistent lesions or complex abscesses, has shown a higher recurrence rate. In an analysis of 120 patients, those who underwent surgery as first-line treatment had a recurrence rate of 66.1%, significantly higher than those treated with steroids alone or in combination with immunosuppressants [16]. This finding suggests that surgery should be strictly reserved for refractory cases, with close postoperative follow-up.

In recent years, intralesional steroids have gained popularity as an effective and less toxic alternative. A systematic review of nine studies including 474 patients demonstrated remission rates comparable to systemic steroids, but with a lower incidence of adverse effects and, in many cases, lower recurrence [17]. This approach offers a viable option for patients with localized disease or comorbidities that contraindicate systemic immunosuppression. An additional study of 78 patients showed that treatment with intralesional steroids combined with topical steroids achieved significantly higher remission rates than oral steroids (93.5% vs. 71.9%), with lower recurrence rates (8.7% vs. 46.9%) and less need for surgery (2.2% vs. 9.4%) [18]. These findings promote the use of local approaches as first-line treatment in selected patients.

Furthermore, the coexistence of systemic manifestations, such as arthritis, has been described in patients with GIM. A case series documented that the use of MTX or AZA not only controlled mastitis but also associated joint symptoms, suggesting a systemic autoimmune nature of the disease in some cases [19]. This finding warrants a comprehensive rheumatologic evaluation in recurrent cases or those with extraparenchymal symptoms.

Even in cases resistant to conventional steroids and immunosuppressants, successful alternative therapies have been described. Mycophenolate mofetil, for example, achieved complete resolution in a patient with IGM refractory to multiple lines of therapy, opening a new avenue for the treatment of severe or resistant forms.

Finally, the identification of *Corynebacterium kroppenstedtii* in breast abscesses has raised the hypothesis of an underlying infectious component. In a series of cases, the targeted use of antibiotics such as teicoplanin allowed remission without the need for immunosuppression, highlighting the importance of performing adequate cultures before initiating steroids, especially in regions with a high prevalence of tuberculosis or chronic infections [20].

Current evidence strongly supports the use of steroids as a therapeutic base for IGM, but cautions that their efficacy depends on the inflammatory phenotype, extent of involvement, and the regimen used. Combined use with immunosuppressants reduces relapses and allows for steroid dose reduction. The intralesional route is emerging as an effective option with lower toxicity. These findings underscore the need for individualized therapeutic strategies and the generation of local evidence to guide decisions in specific contexts such as Mexico.

Predisposition to the development of malignant breast tumors

Regarding oncological risk, there is no direct or sufficient evidence to propose a causal relationship between IGM and an increased incidence of breast cancer [2,4]. It has been suggested that alterations in the breast microbiome and deterioration of the extracellular matrix could be involved in cellular transformation, although these mechanisms remain under investigation [1,4].

Challenges in idiopathic granulomatous mastitis

The diagnosis of idiopathic granulomatous mastitis (IGM) remains a clinical challenge due to its non-specific presentation and the need to exclude other infectious or neoplastic entities before starting immunosuppressive treatment. The prolonged clinical course, variable responses to treatment, and the absence of specific biomarkers make timely diagnosis difficult, which can delay appropriate management and worsen the inflammatory process. In contexts with diagnostic and therapeutic limitations, such as Mexico, the lack of standardized clinical guidelines requires individualized treatment. Evidence suggests that the Mexican population presents a clinical course similar to that reported in other countries, highlighting the need to generate local evidence that allows international therapeutic regimens to be adapted to our clinical and epidemiological conditions [19].

Report limitations

This report presents limitations inherent to its descriptive and retrospective design. Because it deals with only two clinical cases, it is not possible to generalize about the efficacy of steroid treatment in IGM. Furthermore, clinical outcome was assessed qualitatively, without objective scales, which introduces interpretation bias. There were no standardized dosing protocols or defined discontinuation criteria, reflecting both a limitation of the study and of the current clinical approach. Finally, follow-up was limited to the period documented in the medical record, preventing the evaluation of relapses or long-term adverse effects.

Clinical implications and management recommendations in our population

These findings illustrate the heterogeneity in therapeutic response among patients with idiopathic granulomatous mastitis (Table 2). Corticosteroids remain the preferred first-line therapy, while immunosuppressive agents such as methotrexate may be considered in cases of recurrence or corticosteroid resistance. Surgical intervention is generally reserved for persistent or complicated cases. Tailoring treatment to the clinical presentation may help optimize remission rates and reduce recurrences.

**Table 2:** Therapeutic regimens reported in the literature for idiopathic granulomatous mastitis This table was created independently by the authors using data extracted from Montazer et al. [12], Peña-Santos et al. [15], Lermi et al. [16], and Toktaş et al. [18]

Treatment type	Average dose used	Approximate duration	Remission rate (%)	Relapse rate (%)
Oral steroids (prednisone)	0.5–1 mg/kg/day (e.g., 20–50 mg/day)	6–12 weeks or longer	70–80%	30–40%
Tapered oral regimen (Montazer)	50 → 25 → 12.5 → 5 mg every 2 weeks	2 months	93%	0%
With methotrexate (MTX)	MTX 7.5–25 mg/week + prednisone	Induction: 3–6 months	90–95%	<10%
With azathioprine (AZA)	AZA 50–100 mg/day ± prednisone	Maintenance: ≥6 months	95.3%	4.7–9.1%
Intralesional steroids	Triamcinolone 10–40 mg/injection (1–4 sessions)	1–3 months	71.9–93.5%	8.7–46.9%
Antibiotics only (inadequate management)	Dicloxacillin, TMP-SMX (no steroids)	Variable, ineffective	Low (<30%)	High (>60%)
Surgery as first-line treatment	Resection or surgical drainage	—	<60%	66.1%

## Conclusions

These cases contribute significantly to the clinical understanding of granulomatous mastitis by demonstrating the diagnostic complexity when it presents in immunocompromised patients, especially with suspected mammary tuberculosis. Although both entities are rare, their overlap can generate diagnostic uncertainty and delay therapeutic decisions.

The difficulty in distinguishing between idiopathic granulomatous mastitis and mammary tuberculosis directly affects treatment, as the strategies are completely different: while in IGM, steroids can be used to control inflammation, in tuberculosis, starting immunosuppressants without specific antibiotics is contraindicated. This case demonstrates that, without a clear diagnosis, physicians are limited to offering only surveillance, which may not be sufficient if there is an active, untreated infection. It is necessary to maintain a high level of clinical suspicion in patients with risk factors and to remember that not all painful breast lumps are cancer or common infections. It is also essential to promote further research to develop more sensitive and specific diagnostic tests for breast tuberculosis and to establish safe and effective management protocols, especially in vulnerable populations such as immunosuppressed patients.

An additional clinical challenge is the management of women who are actively breastfeeding, where prolonged corticosteroid regimens may raise concerns regarding neonatal exposure. This reinforces the importance of tailoring therapy to the patient's reproductive stage and of generating further evidence in this specific population. Only through rigorous study and clinical reflection can we develop diagnostic tools that not only improve treatment but also provide certainty to those caught between two probable diagnoses and no definitive answer. In cases like this, medicine becomes not only a science but a profound exercise in observation, patience, and compassion.

## References

[1] Krawczyk N, Kühn T, Ditsch N (2024). Idiopathic granulomatous mastitis as a benign condition mimicking inflammatory breast cancer: current status, knowledge gaps and rationale for the GRAMAREG Study (EUBREAST-15). Cancers (Basel).

[2] Barreto DS, Sedgwick EL, Nagi CS, Benveniste AP (2018). Granulomatous mastitis: etiology, imaging, pathology, treatment, and clinical findings. Breast Cancer Res Treat.

[3] Zhu J, Miao X, Li X, Zhang Y, Lou Y, Chen H, Liu X (2024). Granulomatous lobular mastitis co-existing with ductal carcinoma in situ: Report of three cases and review of the literature. Ann Diagn Pathol.

[4] Choi SH, Jang KS, Chung MS (2015). Bilateral granulomatous mastitis with a different etiology. Cancer Biomark.

[5] Pluguez-Turull CW, Nanyes JE, Quintero CJ, Alizai H, Mais DD, Kist KA, Dornbluth NC (2018). Idiopathic granulomatous mastitis: manifestations at multimodality imaging and pitfalls. Radiographics.

[6] Chalmers R, McClellan P, Silva V, Shutt N, Restini C (2020). Red flags for the differential diagnosis of granulomatous mastitis: a case report. J Med Case Rep.

[7] Yin Y, Liu X, Meng Q, Han X, Zhang H, Lv Y (2022). Idiopathic granulomatous mastitis: Etiology, clinical manifestation, diagnosis and treatment. J Invest Surg.

[8] Ravikumar MS, Palanisamy V, Tamilselvan M, Sangili S, Sethuratnam R (2021). Technical aspects: Coronary artery bypass grafting in a case of dextrocardia with situs inversus. Cureus.

[9] Bhattarai P, Srinivasan A, Valenzuela CD, Sulzbach C, Wallack MK, Mariadason JG (2022). Idiopathic granulomatous mastitis: experience at a New York hospital. Ann R Coll Surg Engl.

[10] Gurluler E, Senol K (2025). The recurrence outcome with respect to treatment choices in idiopathic granulomatous mastitis: a retrospective cohort study with 10-year single-center experience. Asian J Surg.

[11] Das Sheth A, Joshi S, Kumar A (2025). Management of idiopathic granulomatous mastitis: effectiveness of a steroid-free regimen using Tinospora cordifolia - a single-institution experience. Breast J.

[12] Montazer M, Dadashzadeh M, Moosavi Toomatari SE (2020). Comparison of the outcome of low dose and high-dose corticosteroid in the treatment of idiopathic granulomatous mastitis. Asian Pac J Cancer Prev.

[13] Gupta N, Vats M, Garg M, Dahiya DS (2020). Bilateral idiopathic granulomatous mastitis. BMJ Case Rep.

[14] Senol K, Ozsen M, Gokalp G, Tolunay S, Tasdelen MI (2025). Efficacy of azathioprine in reducing recurrence in idiopathic granulomatous mastitis. Sci Rep.

[15] Peña-Santos G, Ruiz-Moreno JL (2011). Idiopathic granulomatous mastitis treated with steroids and methotrexate (Articlee in Spanish). Ginecol Obstet Mex.

[16] Lermi N, Ekin A, Ocak T (2023). What predicts the recurrence in ıdiopathic granulomatous mastitis?. Clin Rheumatol.

[17] Vercoe J, Sedaghat N, Brennan ME (2025). Intralesional steroid injections for management of granulomatous mastitis: a systematic review of treatment protocols and clinical outcomes. Breast J.

[18] Toktas O, Konca C, Trabulus DC (2021). A novel first-line treatment alternative for noncomplicated idiopathic granulomatous mastitis: combined İntralesional steroid injection with topical steroid administration. Breast Care (Basel).

[19] Islam S, Stonelake P, John H (2024). OA15 A case series of idiopathic granulomatous mastitis patients and their immunosuppressive management. Rheumatology.

[20] Stevenson DR, Das S, Lambourne J, Ledwidge SF, Johnson L, Rosmarin C (2022). Corynebacterium kroppenstedtii breast abscesses in context, a retrospective cohort study. J Med Microbiol.

